# Navigation, Adoption, and Use of Digital Health Technologies for Irritable Bowel Syndrome Self-Management: Focus Group Study of Patient Experience and Decision-Making

**DOI:** 10.2196/75012

**Published:** 2026-02-02

**Authors:** Adrijana D'Silva, Nicolle Hua, Mary V Modayil, Judy Seidel, Deborah A Marshall

**Affiliations:** 1 Department of Community Health Sciences Cumming School of Medicine University of Calgary Calgary, AB Canada; 2 Primary Care Alberta Edmonton, AB Canada; 3 Department of Medicine Cumming School of Medicine University of Calgary Calgary, AB Canada; 4 O’Brien Institute for Public Health Cumming School of Medicine University of Calgary Calgary, AB Canada

**Keywords:** decision-making, digital health, health literacy, irritable bowel syndrome, mobile applications, patient experiences, patient participation, qualitative study, self-management, technology

## Abstract

**Background:**

Irritable bowel syndrome (IBS) is a common chronic gastrointestinal disorder that impairs bowel functions and patients’ overall quality of life. IBS-focused digital health technologies (DHTs), including online health resources and mobile health (mHealth) apps, have recently proliferated for patient use. However, research exploring patients’ experiences with navigating, adopting, or using commercial or publicly available DHTs for IBS self-management is limited.

**Objective:**

This study aims to explore the user experiences and decision-making of patients with IBS as they navigate, adopt, and use diverse DHTs for disease self-management.

**Methods:**

We conducted virtual semistructured focus group interviews to explore the experiences of patients with IBS using DHTs, including their perspectives on design and features, their decision-making process in using DHTs, and recommendations for improving user experience and uptake, given the heterogeneous nature of these tools. Canada-based patients with IBS who were using or had used mHealth apps to manage symptoms were recruited through purposive sampling from previous IBS-related studies. Discussions were transcribed verbatim, and inductive thematic analysis was performed using NVivo (version 14; Lumivero). A modified version of the Expanded Unified Theory of Acceptance and Use of Technology (UTAUT2) model was applied to guide the interpretation of the dynamic relationship between the influences on participants’ decisions regarding DHT use.

**Results:**

Among the 8 participants (all female; mean age 55.3, SD 13.5 years), two themes were identified: (1) uncertainty impacts the trustworthiness of DHTs, and (2) influences that drive the decision-making process to adopt and use DHTs. The observed influences aligned with the constructs of the UTAUT2 model (performance expectancy, effort expectancy, social influence, facilitating conditions, hedonic motivation, price value, and habit), with the addition of trust and risk in participants’ decision-making. Digital health literacy and patient engagement were also raised as crucial components of participants’ experiences and perspectives on DHTs.

**Conclusions:**

Findings of this study highlight the current landscape of digital health in IBS and existing gaps and challenges for patients in navigating, adopting, and using DHTs for IBS self-management. While DHTs were generally viewed positively for their value and potential, patients with IBS consider several coexisting factors and trade-offs in their decision-making. Further investigations on the influences on and perspectives toward DHTs could enhance future development and iterations of these tools and improve patient confidence and uptake.

## Introduction

Digital health has revolutionized health care and disease management by leveraging technology and digital devices to improve self-management. With a growing public focus on health and well-being, more patients with chronic diseases rely on digital health, especially online health resources and mobile health (mHealth) apps [1,2], for answers, guidance, and management support.

Irritable bowel syndrome (IBS) is a disorder of the brain–gut connection that impacts patients’ daily activities and well-being and is characterized by abdominal pain, bloating, altered bowel habits, and psychiatric comorbidities [3-5]. As there is no known cure for IBS, greater knowledge of the syndrome and relevant self-management options are critical in alleviating symptoms and improving quality of life [6,7]. However, IBS self-management strategies—such as lifestyle and dietary modifications and brain–gut behavior therapies [8,9]—are often coupled with challenges around accessibility, adherence, affordability, and productivity loss for patients [10-12]. Digitizing health information and support could offer alternative models of delivery and potentially address these issues by improving access to information and treatments, reducing health-related costs, and enabling and empowering patients [13-16].

In the context of IBS, digital health technologies (DHTs) encompass a diverse range of tools that support dietary, psychological, behavioral, and educational aspects of self-management. Commonly used dietary-focused tools include low fermentable oligosaccharides, disaccharides, monosaccharides, and polyols (FODMAP) diet apps, which are supported by evidence for improving symptom control and dietary adherence [17]. Psychological and brain–gut behavior apps have also demonstrated effectiveness in reducing IBS symptom severity and improving quality of life [18,19]. In addition, patients frequently use general symptom trackers, medication apps, and online educational resources of varying quality. This heterogeneity underscores the importance of understanding not only whether patients use DHTs, but also the specific domains these tools address, as each represents a difference in therapeutic mechanisms and levels of evidence within IBS care.

Our understanding of DHTs for IBS remains limited. Although several studies support the benefits of DHTs for patients with IBS [20], there is limited evidence on their experiences in a consumer context. Existing studies that qualitatively evaluate DHTs, although informative, have been limited to specific DHTs under controlled conditions [21-23] and otherwise lacked the sociodemographic context that could otherwise impact uptake and use. Other studies suggest acceptance of DHTs is determined by users’ hedonic motivations, user-system fit, and perceived utility and intuitiveness of the technology [24-26]. However, it is uncertain how these influences or other priorities influence this specific patient cohort. Currently, it is difficult to assess other considerations and consequences for the real-world application of DHTs for the people with IBS. This study is an initial investigation to explore the experiences of patients with IBS in navigating, adopting, and using online health resources and mHealth apps to support self-management, and to identify key influences in their decisions to consider DHTs.

## Methods

### Ethical Considerations

This study was reviewed and approved by the University of Calgary Conjoint Health Research Ethics Board (REB23-1273). A qualitative study design with semistructured focus groups was used to explore the experiences and decision-making processes of patients with IBS regarding DHTs, including their adoption, use, and suggestions for improving these tools. The study methodology was reported in accordance with the COREQ (Consolidated Criteria for Reporting Qualitative Research) checklist (Multimedia Appendix 1) [27].

### Positionality

The study team comprised 5 women with research experiences in health systems (AD, NH, MVM, JS, and DAM), including one (AD) with lived experiences of IBS. AD and NH, a postdoctoral fellow and research associate, respectively, were skilled in qualitative methods. AD and DAM, a health economist and health services researcher, also had experience in IBS-related research. MVM and JS, a senior scientist and scientific director, respectively, had applied research expertise and experience in primary care. All but one each have a PhD; NH has a master’s degree. Information on AD’s personal experience and professional background in IBS was shared with study participants prior to the focus group sessions. Some participants had an existing relationship with AD from previous research studies.

### Participants

Participants were recruited through purposive sampling from previous IBS-related studies conducted by the study team. Past participants who had provided consent to be recontacted for future studies were invited by email. Convenience sampling was attempted using the study team’s social media networks and the University of Calgary’s research recruitment board; while 2 individuals were recruited through this method, neither participated in the focus groups. All participants were adults with IBS living in Alberta and British Columbia, Canada, who (1) had been formally diagnosed by a health care professional and (2) had been using or had used mHealth apps to manage their symptoms. Participants who satisfied the selection criteria received an information document regarding the study and provided written consent before the focus group sessions.

The study team a priori aimed for at least 4 focus groups, with 3 participants per group, which was expected to be sufficient to reach code saturation [28-30]. While focus group size typically ranges from 6 to 8 participants, smaller groups with 3 participants were considered to allow more opportunities for engagement and account for the potentially low recruitment yield [28]. The recruitment process ended following the exhaustion of all recruitment strategies, resulting in 8 participants and 3 focus groups. Some degree of saturation was present in participants’ responses through repeated codes and themes.

### Data Collection

Participants completed a survey before attending the focus group session to provide details of their existing supports, demographic information, year of IBS diagnosis, symptoms, treatment history, and dietary adjustments (Multimedia Appendix 2). The survey and data were managed using Qualtrics Experience Management (Qualtrics LLC) and the University of Calgary’s secured online cloud server. Survey data were analyzed and summarized using Microsoft Excel by Darren Zhang.

Focus groups were conducted virtually using Microsoft Teams. AD moderated the focus group discussions, with the support of NH as a scribe. Sessions lasted approximately 2 hours, were audio-recorded using the recording feature in Microsoft Teams, and were transcribed verbatim using the transcription service Rev (Rev.com, Inc); the transcripts were subsequently reviewed and cleaned by Darren Zhang.

### Instrumentation

Participants’ IBS symptoms were assessed in the survey using the IBS Symptom Severity Score (IBS-SSS); scores ranged from 0 to 500 and were categorized as mild (75-175), moderate (176-300), or severe (>300) [31]. A focus group guide was created and reviewed by the study team to support AD in facilitating semistructured sessions (Multimedia Appendix 3). The following topics were discussed in the focus groups: (1) participants’ experiences navigating digital resources to self-manage IBS, (2) their experiences adopting and using DHTs to self-manage IBS, and (3) their perspectives on influences and improvements regarding the uptake of online resources and mHealth apps.

### Data Analysis

Coding was performed and managed using NVivo (version 14; Lumivero). NH and AD coded the transcripts as primary and secondary coders, respectively. Because little research has explored this topic, inductive thematic analysis was used to identify and evaluate relationships, patterns, or themes, allowing data to serve as the foundation of the results [32]. Initial codes were generated to indicate recurring features within participants’ responses, and related codes were then collated into groups that subsequently defined underlying themes [32]. Following thematic analysis, the findings and their relationships were visualized based on the Expanded Unified Theory of Acceptance and Use of Technology (UTAUT2) framework model [26].

### Applied Framework

The UTAUT2 framework model is an extended version of the original UTAUT model to evaluate the acceptance of new commercial technologies [26,33]. With its consumer-use context, this model was chosen to provide valuable and holistic insight into patients’ acceptance of DHTs, including adoption and use, for IBS self-management. The key constructs of the UTAUT2 model [26] included performance expectancy, defined as the extent to which the individual expected the performance or benefits of the technology; effort expectancy, defined as the level of effort or ease in using the technology by the individual; social influence, defined as the extent to which the individual perceived the value of others’ acceptance of the technology; facilitating conditions, defined as the extent to which the individual believed that support or resources were available to facilitate the use of the technology; hedonistic motivation, defined as the extent of the individual’s motivations or pleasures experienced in using the technology; price value, defined as the cost-benefit or trade-off between the perceived value or benefit of the technology and its associated monetary cost; and habit, defined as the extent to which the individual developed a habitual or perpetual use of the technology from previous experience and learning.

Also, we modified the UTAUT2 model to include “trust” and “risk” as key constructs based on our thematic findings. Although they were not included in the original UTAUT2 model [26], previous studies have recognized and incorporated them into their own models [34-36]. Based on these studies [34-36], “trust” was defined as the extent to which an individual perceived the technology or its associated qualities as likable or trustworthy, and their willingness to believe that the technology would meet their expectations, and “risk” was defined as the extent to which an individual perceived the technology to be associated with potential consequences or harm to themselves.

Additionally, DHT-related key constructs are influenced by user characteristics. In the expanded UTAUT2 model, Venkatesh et al [26] recognized how a user’s demographic characteristics, specifically age, gender, and experiences or familiarity of existing technologies, moderate the impacts of the key constructs and ultimately the acceptance and use of the technology.

## Results

### Participant Characteristics

Of the 11 participants recruited to the study, 8 attended the focus groups, all of whom had participated in past research studies. One individual was lost to follow-up prior to the focus group sessions, and the remaining 2 could not attend the sessions due to scheduling conflicts. Participant characteristics are summarized in Table 1.

**Table 1:** Participants’ characteristics (N=8).

Characteristics			Value
Age (years), mean (SD)			55.3 (13.5)
Gender (female), n (%)			8 (100)
**Highest education, n (%)**			
	Postsecondary certificate or diploma below bachelor level or apprenticeship or trades certificate	1 (13)	
	Bachelor’s degree	1 (13)	
	Postsecondary certificate or diploma above bachelor level	4 (50)	
	Master’s degree	2 (25)	
**Identify as, n (%)**			
	Born outside of Canada	3 (38)	
	Persons with disabilities	2 (25)	
	2SLGBTQI+^a^	1 (13)	
	Racialized or visible minority	1 (13)	
	Indigenous	0 (0)	
	I have children or grandchildren aged 18 years or younger living at home	0 (0)	
	New to Canada (less than 5 years)	0 (0)	
	Other	1 (13)	
	None of the above	3 (38)	
**Years since IBS^b^ diagnosis, mean (SD)**			12.9 (10.6)
**Types of IBS, n (%)**			
	Mixed	2 (25)	
	Constipation-dominant	5 (63)	
	Diarrhea-dominant	1 (13)	
	Unsure	0 (0)	
IBS-SSS^c^, mean (SD)			197.7 (84.8)
**Types of digital tool used, n (%)**			
	Mobile health apps	7 (88)	
	IBS-related podcasts	4 (50)	
	Mobile tracking apps (eg, physical activity and daily living activities)	6 (75)	
	Websites	6 (75)	
	Support groups	1 (13)	
**Types of mobile health apps (n=7), n (%)**			
	Diet-related apps	2 (29)	
	IBS-specific apps	6 (86)	
	Mental health or emotional well-being apps	1 (14)	
	General gastrointestinal and health apps	2 (29)	
	Cognitive-behavioral therapy and/or gut hypnotherapy apps	1 (14)	

^a^2SLGBTQI+: two-spirited, lesbian, gay, bisexual, transgender, queer/questioning, intersex, and others.

^b^IBS: irritable bowel syndrome.

^c^IBS-SSS: IBS Symptom Severity Score; ranges from 0 to 500, with mild (75-174), moderate (175-299), and severe (300-500) symptom severity.

All participants identified as women and had tertiary-level education. The mean age was 55.3 (SD 13.5) years, and participants had lived with an IBS diagnosis for an average of 12.9 (SD 10.6) years. Five of 8 (63%) participants reported constipation-dominant IBS, 2 (25%) reported mixed IBS, and 1 (13%) reported diarrhea-dominant IBS. The average IBS-SSS was 197.9 (SD 84.8), indicating mild symptoms. Participants had managed their IBS symptoms using one or a combination of the following DHTs: a total of 7 (88%) had used mHealth apps, 6 (75%) used mobile tracking apps, 6 (75%) used websites, 4 (50%) used IBS-related podcasts, and 1 (13%) used support groups. Among all types of mHealth apps used (n=7), 6 participants (86%) reported having used IBS-specific apps, 2 (29%) diet-specific apps, 2 (29%) general gastrointestinal and health apps, 1 (14%) mental health and/or emotional well-being apps, and 1 (14%) cognitive-behavioral therapy and/or gut hypnotherapy apps.

### Themes of Navigating, Adopting, and Using Digital Health Technologies

Two themes were identified from participants’ experiences and decision-making around navigating, adopting, and using DHTs for IBS self-management: (1) uncertainty impacts the trustworthiness of DHTs, and (2) underlying influences drive the decision-making process to adopt and use DHTs.

#### Theme 1: Uncertainty Impacts the Trustworthiness of Digital Health Technologies

A sense of uncertainty was prevalent among participants when navigating, adopting, and using DHTs for their symptoms of IBS. While participants were generally positive toward DHTs with hope and expectations of effective self-management and symptomatic relief, the coexisting challenges and risks contributed to varying levels of distrust.

Participants expressed uncertainty toward the effectiveness, utility, and understanding of DHTs. Because of the self-directed nature of the navigation process through their mobile devices and the internet, participants were aware that not all DHTs would be effective or suitable for them, and some recounted their experiences of finding suitable DHTs through trial-and-error.

[ . . . ] As we know, it is hard to know if anything's working. It's hard to know if anything works for the symptoms that we're experiencing. So that does come into it as well, but it's not a science, it's sort of a bit of a feeling about whether you're going to continue with something and whether it's worth it.

Participant #48

I just found and tried and listened to whatever. And then if it wasn't in my wheelhouse then I just discarded.

Participant #29

In contrast to those with positive experiences, other participants with subpar experiences using DHTs expressed challenges and frustration in finding what they needed to manage IBS. The lack of guidance around appropriate and trustworthy DHTs left some feeling alone and overwhelmed by the amount of information online or the number of mHealth apps to consider.

We just kind of have to search and go online and try to look at reliable resources [ . . . ] I've got family help too that they're in the tech side of things, so I get some help from them from looking at different options. But basically, you do it on your own.

Participant #37

You're feeling crummy. You need a resource, you need some help, but trying to wade through everything or do a Google (search) is just so overwhelming.

Participant #9

Uncertainty toward the DHTs’ intentions was raised, including fear of scams or malicious attacks from illegitimate websites and privacy and data exploitation from mHealth apps. Furthermore, participants discussed the accuracy of online information from the internet and their concerns about consuming IBS-related misinformation and disinformation.

I agree with (Participant #9) regarding credentials and if (doctors are) trying to sell stuff. I know there's some doctors out there who do that too. So yeah, just mindful of people trying to market products versus people with credentials who are trying to debunk misinformation or provide just evidence backed information.

Participant #14

I don't like even giving my credit card information or any other personal information out into the internet. You just never know who's out there and what's going to happen to it.

Participant #37

During these discussions, digital health literacy was raised as an essential skill and a common barrier for patients with IBS to assess, process, and understand digital health information and technologies.

It's not about (like) or dislike, it's always questioning what I found, is it true or not, and how to explore further to be sure that these things are true because I'm not a great researcher [ . . . ] because of lack of medical background. So I am always hesitant to trust this or that information. It's not disliking, but it's a concern whether it's right direction to move or not.

Participant #4

#### Theme 2: Influences That Drive the Decision-Making Process to Adopt and Use Digital Health Technologies

##### Overview

**Figure 1:**
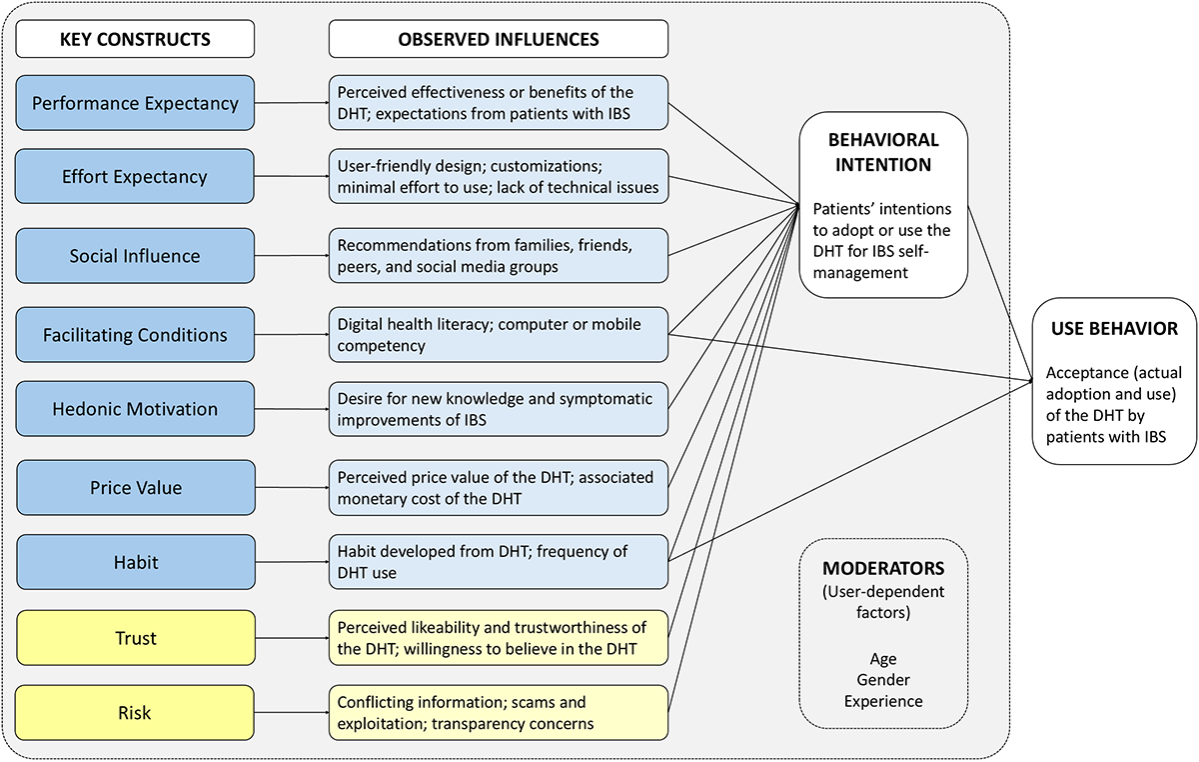
Relationships of identified influences in the decision-making process of patients with irritable bowel syndrome (IBS) on the use and acceptance of digital health technologies (DHTs) based on the modified Expanded Unified Theory of the Use and Acceptance of Technology (UTAUT2) model. “Trust” and “risk” were not part of the original UTAUT2 model and were included as a result of the study’s findings.

Participants alluded to several influences that contributed to their decision to use DHTs, which were contextualized using the modified UTAUT2 model (Figure 1). Applying the model to this cohort illustrates the model’s relevance and the complexities of DHT acceptance among patients with IBS. The observed influences (key constructs), as proponents or opponents to their decision-making process at varying degrees, collectively impact participants’ intentions and/or the actual adoption or use of DHTs. Furthermore, the influences may have been externally moderated by participants’ demographics, including age, gender, and experience or familiarity with DHTs. Selected quotes are provided to illustrate the influences and their relation to key constructs from the UTAUT2 model (Multimedia Appendix 4).

##### Performance Expectancy

Based on their prior knowledge, participants expressed varying levels of preconceived expectations toward the DHTs’ performance. Aspects of participants’ knowledge prior to using the DHTs included past experiences and associated emotions, user-system fit, health and disease knowledge, social influences and recommendations, and preexisting familiarity or trust toward the tool. Most participants therefore evaluated the performance of mHealth apps based on one or multiple outcomes: (1) the perceived severity and/or frequency of IBS symptoms before and after use, (2) changes to overall mood before and after use, (3) feelings toward the DHT, and (4) the ability of addressing concerns specific to the user. Online health resources, many of which served to disseminate disease knowledge and information, were evaluated differently by participants compared with apps. Participants instead focused on the accuracy, readability, accessibility, and perceived value of the information when assessing performance. One participant also noted the expectation-reality discrepancy among peers, contrasting high and unrealistic expectations of a fast, curative “fix” with DHTs designed for regular, long-term disease management.

##### Effort Expectancy

Most participants emphasized the importance of user-friendly DHT designs in successfully maintaining user engagement. Some described their experiences with poorly designed mHealth apps as frustrating or overwhelming (eg, rigid features, complicated functions requiring more user effort), and were subsequently discouraged from using the app further or to its fullest potential. Poor user design included features such as health monitoring functions requiring frequent data inputs or stringent timing, lacking customizable functions for personalization, and features with technical difficulties or lacking updates to address bugs. Conversely, apps with a more simplistic and seamless design reducing user effort, along with more customization capabilities, were viewed favorably. One participant considered it a priority to improve patient-centered aspects in DHT designs.

Participants unanimously agreed that patient engagement is critical for DHTs to ensure they are relevant and well-designed to address the needs of patients with IBS. Some participants considered that patients could engage in varying stages of development, such as the design and testing phases. The unanticipated burden of incorporating DHTs into their lifestyles—despite their intended use to improve patients’ quality of life—further underscores the need for patient partners in the health research and technology space.

##### Social Influence

Many participants acknowledged their social circles as valuable support for their IBS diagnosis and management. They specified the type of support they received from their family and friends, specifically in navigating online resources, accessing the appropriate health care professionals, and recommending mHealth apps. However, some felt that certain DHT recommendations from their social circles were unhelpful or irrelevant to their circumstances because of limited knowledge or understanding of IBS. Others also were hesitant to fully embrace their support altogether, citing the social stigma around the condition.

In contrast, peer support groups were viewed favorably as a knowledgeable resource because of shared lived experiences with IBS. Most participants sought IBS support—often through social media platforms—for information regarding IBS-specific professional support, symptomatic management strategies, tools, and resources. One participant explained that peer recommendations could also help patients with IBS in decision-making about using mHealth apps by considering aspects or barriers from another perspective, such as evaluating the price relative to its value or benefits.

##### Facilitating Conditions

Participants’ level of digital health literacy and competency with a device (eg, computer or mobile device) were identified as facilitating conditions contributing to user experiences with DHTs. Participants recognized the importance of digital health literacy in navigating, processing, and critiquing online health information. Many participants were considered to have higher digital health literacy because of their educational or professional backgrounds. Participants also acknowledged the role of computer and mobile competencies required for accepting and using DHTs, along with the significant learning curve for digital competency among older populations.

##### Hedonic Motivations

Most participants mentioned that their decision to adopt and use DHTs stemmed from a desire and willingness to learn about IBS and emphasized the importance of new and updated information available on these tools. Some participants had thoroughly researched and exhausted common IBS-related recommendations; DHTs that regularly provided new content were highly desirable and therefore maintained user engagement. Conversely, most participants struggled to fully engage with DHTs that lacked new content or variety, as they no longer felt the need to continue using these DHTs. Some participants also noted their interest in other IBS topics areas, such as mental health and community engagement.

##### Price Value

Participants frequently evaluated the cost-benefit trade-offs of DHTs—the monetary cost relative to their perceived values and benefits—in their decision-making process. Most participants first opted for services or apps that were free to download and use, but some also considered DHTs with paid business models. However, DHTs following a subscription business model were perceived by participants as the least desirable, with the accrued cost to maintain full access outweighing the potential benefits.

##### Habit

Participants correlated their habit or frequency of using DHTs with the DHTs’ perceived utility, with greater frequency and established positive habits to be associated with symptomatic improvements and user satisfaction. Conversely, DHTs that were seldom used were deemed less useful, thus discouraging participants from using them even further.

The habit of DHT use depended on the features available, specifically reminders, and the digital platform. Some participants noted that customizable reminder functions prompted more consistent engagement from their experiences and were considered especially useful for certain DHT characteristics (eg, symptom and diet tracking and exercise programs); however, one participant mentioned that they did not need to be reminded to use their DHTs. Participants agreed that, given their frequent use of mobile devices (eg, smartphones), mHealth apps are more advantageous in maintaining user engagement. In contrast, participants perceived online websites or programs differently; they were viewed as cumbersome to locate, input login credentials, and search for the content. The integration of lifestyle with digital devices served as an antecedent factor that influenced participants’ level of DHT acceptance and engagement.

##### Trust

In the context of the UTAUT2 model, trust in a DHT influences users’ intention or willingness to use it and subsequently, whether the DHT is used for IBS (Figure 1). When navigating online health resources, participants positively associated medical or academic credibility with trust. Participants often referred to reputable medical websites (eg, Mayo Clinic and WebMD) for health information to improve their knowledge and IBS self-management. Academic and research institutions were also viewed favorably, and the involvement of a credible institution in the development of DHTs heavily weighted in favor of participants’ decision-making. Some participants also acknowledged digital health literacy as a contributing factor toward trustworthiness of DHTs, as the ability to critically assess the information and sources provided greater confidence in recognizing credible websites and apps and placing trust in them.

##### Risk

In the acceptance and use of DHTs, participants weighed the potential risk associated with using DHTs as part of the trade-off with perceived benefits. Conflicting information, transparency concerns, and fear of financial and data exploitation were commonly raised by participants when navigating or using DHTs. For mHealth apps, concerns were primarily related to the handling and security of personal and health information. Regarding online resources, participants recognized the prevalence of conflicting information on the internet and the potential risks posed to unsuspecting users seeking clinical guidance. Participants also recounted their experiences with suspected scams, specifically websites that offered paid products and services to allegedly treat or cure IBS. One participant correlated their familiarity with the DHT and the DHTs’ risk, associating less familiar DHTs with lower trustworthiness and greater risk.

## Discussion

### Principal Findings

#### Overview

Our findings highlight the importance of certain characteristics from both DHTs and users with IBS in determining intentions and success in navigating, adopting, and using DHTs, with many attributes aligning with the UTAUT2 framework [26]. Participants expressed an overarching theme of uncertainty towards DHTs in their abilities to ameliorate symptoms, which is based on their levels of and balance between perceived trust and risk. Other relevant user-specific attributes include digital health literacy and digital competency (facilitating conditions), desire for new knowledge (hedonic motivation), habitual use of DHTs (habit), and recommendations from social circles (social influence). DHT-specific characteristics include considerations around the design and features that influence perceived effectiveness (performance expectancy), user experience (effort expectancy), and value (price value). These considerations holistically illustrate the experiences and perspectives of patients with IBS using DHTs and their underlying and multifaceted influences. Addressing barriers and leveraging facilitators is essential for developing effective DHTs, improving user experience and health outcomes, and promoting innovation within the digital health care space [37-39].

#### Building Trust and Addressing Uncertainties of Digital Health Technologies

Trust is a juxtaposing force to fear of uncertainty and a critical component of successful implementation, adoption, and use of DHTs. However, the unprecedented evolution of the digital health landscape poses several challenges, including limited regulations, guidelines, and a lack of robust evidence base for efficacy, safety, and impact [40,41]. Furthermore, other limitations (eg, technological, costs, and transparency concerns) add to the DHTs’ “black box” nature, which could discourage users from accepting or engaging with DHTs when they are not being fully informed.

In a 2018 scoping review, trust in digital health systems was influenced by several elements, including quality, efficiency, self-efficacy, accessibility, reputation and users’ recommendations, and fear of data exploitation [42]. Many of these enabling and inhibiting elements of trust align with the key constructs of the applied UTATU2 model, further highlighting the complexity and interconnectedness of patient trust in adopting and using DHTs. Participants’ trust in DHTs was also compromised by their fear and the potential risk of harm through scams, malicious digital attacks, and exploitation. Similarly, Catapan et al [43] found commercial and data exploitation concerns to undermine the trustworthiness of digital health in a cohort of patients with chronic kidney disease.

Participants’ hesitancy toward DHTs must also be understood within the broader digital misinformation landscape. Research has shown that IBS-related content on online platforms, such as YouTube and TikTok, is made accessible in the absence of a vetting process, and can be inaccurate, promotional, or lack scientific grounding [44,45]. Likewise, online dietary guidance for IBS often suffers from poor readability and inconsistent quality [46]. Patients with chronic, fluctuating symptoms such as IBS might be particularly vulnerable to online “cures,” unregulated wellness products, or misleading health claims [47]. In this context, skepticism reflects an adaptive response to navigating oversaturated but often unreliable digital health information. Recognizing health misinformation as an external driver of uncertainty reinforces the need for credible and evidence-based DHTs that patients can trust.

Reducing the uncertainties of DHTs and improving patient trust require a large-scale, coordinated, multidisciplinary approach involving technology developers, patients, researchers, health care professionals and institutions, and government bodies. First, improving the technological systems and processes of DHTs for quality, data security, and transparency is needed, with patients and researchers involved to assess effectiveness, utility, value, and feasibility [38]. Second, improving health care system infrastructures to better respond and adapt to DHTs will further facilitate general trust in DHTs among patients and health care professionals [38]. Lastly, stronger regulatory practices and guidelines should be implemented to improve transparency by ensuring that commercial and publicly available DHTs meet efficacy, safety, and privacy standards. While governments have the capacity and means to implement laws and regulatory measures, professional health associations and bodies could also play a pivotal role in supporting health care professionals in navigating and using DHTs alongside their patients. For example, Torous and Roberts [48] argued for the American Psychiatric Association to provide digital health–related resources and guidelines to patients and clinicians. However, while advancing transparency and patient trust is crucial, it is also important to avoid overregulation, which could subsequentially limit or delay DHT innovation, adoption, and use [48].

#### Existing Gaps in Digital Health Literacy

Digital health literacy is a factor in participants’ decision-making on adopting or using DHTs (facilitating condition) and a crucial determinant of health—especially in one’s ability to navigate and critically assess health information online and leverage the benefits of effective DHTs [49]. Our sample was highly educated, which may have influenced their ability to evaluate the quality of online sources effectively without additional support. However, digital inequality is an ongoing issue; patients with lower levels of digital health literacy are more likely to struggle with navigating and using DHTs, considering that high levels of reading comprehension are expected [39,46,50]. Limited understanding of content, benefits, and risks in adopting health technologies hinders patients’ ability to make informed decisions about DHTs, and by extensions, to fully trust them.

Improving digital health literacy requires a collaborative approach involving patients, health care professionals, and policymakers. Strategies include the availability of educational or digital health literacy programs [51] and adapting online information and resources to a broader audience [52]. Technology developers should also tailor their content and design based on the target users’ characteristics, such as digital health literacy levels, and improve transparency around privacy and security to mitigate harm and build trust. By facilitating the conditions in which patients are equipped with the skills and confidence to effectively use DHTs, they could fully leverage these available resources.

#### The Financial Barrier to Digital Health Technologies

Unsurprisingly, affordability was raised as a common barrier for participants to access support, especially mHealth apps with paid models or in-app purchases. While paid apps may be perceived more positively than free apps [37], affordability, or the lack thereof, could impact patients’ adherence to them [53]. In addition to the existing socioeconomic burdens of IBS [54], patients could be especially more conscious of the financial trade-off of adopting and using cost-associated DHTs. Participants’ preferences for one-time payment models over subscription models aligns with similar findings from a patient cohort with rheumatoid arthritis [55], suggesting that certain payment models for DHTs could also influence patients’ decision-making process. Although Xie et al [56] suggested technology developers consider subscription plans to improve affordability and users’ willingness to pay, this approach might instead deter patients with chronic diseases requiring sustained DHT use. More research is warranted to better understand the preference of patients with IBS for DHTs with certain business models to ensure accessibility and acceptance.

#### The Value of Patient Engagement in Digital Health Technology Development

Content and user-centered designs were important aspects of DHT acceptance and use [57]. As part of their hedonic motivations, participants valued new and varied content in DHTs and considered limited and outdated content as a major deterrent. Health information–seeking behaviors are prevalent in people with IBS [58], which could be attributed to a desire and hope for relief or a cure [47]. Furthermore, IBS is a complex disorder involving individualized brain–gut interactions and requires personalized medicine [9]. As a result, the accuracy, variety, and customizability of the content are considered invaluable for patients to explore self-management strategies from a multidisciplinary perspective [59].

User-centered design is an iterative and collaborative process that requires a foundational understanding of the target users and the context of their intended use [60]. The outcome of such design is heavily dependent on comprehensive research and observations, feedback, and recommendations from lay individuals and experts at all stages of development (eg, initial design, evaluation, and user feedback) [60,61]. Patient engagement in digital health research and development is, therefore, fundamental to constructing a successful user-centered design in DHTs for optimal efficacy and impact [61].

However, patient engagement remains limited in digital health and technology innovation [62], given existing challenges around digital health literacy, accessibility, and trust and transparency concerns [39]. Careful planning and considerations are also needed to leverage patients’ inputs effectively; patients are most often involved in usability testing, where DHTs are mostly finalized which consequently leaves little flexibility to implement feedback [62]. Although there has been encouraging efforts to further engage patients with IBS in digital health research and implementation science [63], more is needed to fully ascertain the implications of DHTs and develop robust evidence specific to this population group.

### Strengths and Limitations

This study is the first to explore the experiences of patients with IBS in navigating the digital landscape for self-management using the UTAUT2 model. Our findings contribute to the limited existing literature by providing insight into how DHTs can support IBS symptom management while addressing concerns related to distrust and digital literacy. We also identified key barriers and facilitators that impact patient experience and outcomes and provide recommendations for improvement based on patient perspectives.

This study also has several limitations. The transferability of our findings may be limited due to the small sample size and the presence of self-selection and sampling bias. First, our recruitment method used purposive sampling, where individuals decided whether to participate in the study. Our sample was highly educated, demonstrated high levels of digital health literacy, and showed great interest and motivation in health research and therefore was not representative of the general population with IBS. Second, all participants identified as women, which may be due to a combination of potential gender-related differences in IBS prevalence [64], mHealth app use [65], or the gender disproportion of the recruitment pool from previous IBS studies. This, however, poses the challenge of evaluating gender-based differences in this study, especially when gender is a moderating variable in both the original and expanded UTAUT models [26,33]. Third, all but one participant lived in a single Canadian province; their access or experience with health services and other non-DHT support may be different from other regions of Canada or worldwide. Lastly, coding saturation could not be fully attained in this study due to the insufficient sample size. While we have identified the most prevalent themes, having additional focus groups would further capture the themes comprehensively [30]. This would also avoid data bias and ensure the content validity, replicability, and transferability of the study [66]. Nevertheless, this study achieved its aim of building a deeper understanding and appreciation of patient experiences, perspectives, and decision-making around DHTs used for IBS that could serve as an antecedent for future studies and a guide in developing more patient-centered resources and supports.

### Future Directions

Given the central role of digital literacy in our findings, future research should prioritize developing and evaluating educational or digital literacy programs specifically designed for patients with IBS. Identifying the unique barriers these patients face can guide the development of effective educational interventions. Additionally, longitudinal studies examining how enhanced digital literacy impacts the adoption and sustained use of digital tools would be valuable.

An emerging aspect in digital health is the rapidly expanding role of artificial intelligence and large language models (LLMs), such as ChatGPT, in supporting patient decision-making. Early evaluations suggest advanced LLMs can provide clinically aligned guidance, summarize complex medical concepts, and support initial health information–seeking more effectively than traditional search engines [67-69]. For patients with IBS, LLMs could serve as navigational tools that help filter misinformation, translate evidence-based information into patient-friendly language, and direct users toward credible DHTs or reputable resources. At the same time, concerns regarding bias, hallucinations, privacy, dependence on the quality and reliability of available training data and prompts, and lack of transparent regulatory oversight highlight that these tools remain imperfect and require careful integration into IBS self-management [70-72]. Because LLM outputs are only as trustworthy as the data and sources on which they are trained, they may inadvertently reproduce inaccuracies or amplify misinformation. As AI-driven tools become more embedded in digital health, understanding how patients with IBS engage with them and how these tools can be responsibly implemented will become a critical future next step in this research. As AI-enabled tools and LLMs become increasingly integrated into consumer-health information-seeking behaviors, future work should also examine how these systems can support digital health literacy and help patients with IBS navigate an often-confusing online environment. Understanding how patients with IBS engage with these emerging technologies will be essential for designing safe and supportive digital self-management tools.

The evidence base on the feasibility and implementation of digital health technology for IBS is still lacking but necessary for real-world application. Understanding certain DHT features and attributes in patients’ decision-making process could serve as a guide for future research and projects to understand and prioritize key objectives. Comprehensive economic evaluations should consider the direct and indirect costs of adopting DHTs in patients’ self-management practices, as cost was considered a major barrier among participants.

Another fundamental step in this field is to improve patient engagement in health research and technology development. User-centered design was highlighted as a crucial component for the success of DHTs, and the need for greater involvement of patients in developing DHTs. Patient partners in future research and commercial development of DHTs, especially in the design and testing phases, will help ensure these tools are intuitive and meet patients’ needs. Patient engagement will also be valuable in providing opportunities to quantify the significance of the identified influences in this study; this information could offer further insight into patients’ decision-making process and how they weigh their considerations holistically.

### Conclusion

This study highlights the complexities of the digital landscape in the context of IBS. While there is optimism toward DHTs in enhancing self-management, concerns about digital health literacy, trustworthiness, access, and cost must be addressed. With user-friendly, accessible, trusted, and cost-effective tools, the effectiveness and adoption of digital health solutions can be enhanced, and the digital health user experience optimized. It is evident that integrating DHTs in IBS self-management is a multifaceted, complex task that requires comprehensive and interdisciplinary collaboration among technology developers, health services, health professionals, and patients. More efforts are needed to address the identified barriers and leverage facilitators to improve health outcomes and quality of life for patients managing IBS or other chronic diseases.

## Appendix

Multimedia Appendix 1 COREQ checklist.

Multimedia Appendix 2 Pre-focus group survey of participants’ characteristics, IBS severity, and digital health tools used for IBS self-management.

Multimedia Appendix 3 Semistructured focus group interview guide.

Multimedia Appendix 4 Participant quotes of observed influences in considering DHT adoption and use for IBS patients in relation to key constructs based on the Expanded Unified Theory of Acceptance and Use of Technology (UTAUT2) model.

## Abbreviations

COREQ: Consolidated Criteria for Reporting Qualitative Research
DHT: digital health technology
FODMAP: fermentable oligosaccharides, disaccharides, monosaccharides, and polyols
IBS: irritable bowel syndrome
IBS-SSS: IBS Symptom Severity Score
LLM: large language model
mHealth: mobile health
UTAUT2: Expanded Unified Theory of Acceptance and Use of Technology

## Data Availability

The datasets generated or analyzed during this study are not publicly available, as participants did not provide consent to share their individual data.
