# Supplementary material for: Navigation, Adoption, and Use of Digital Health Technologies for Irritable Bowel Syndrome Self-Management: Focus Group Study of Patient Experience and Decision-Making
Source: JMIR Hum Factors. 2026 Feb 2;13:e75012. doi: 10.2196/75012 (PMC12910269; doi:10.2196/75012)
Supplement: Multimedia Appendix 2 [file humanfactors_v13i1e75012_app2.doc]

## **Multimedia Appendix 2:** Pre-focus group survey of participants’ characteristics, IBS severity, and digital health tools used for IBS self-management.

## **Pre-Focus Group Survey**

Thank you for joining this study. In this survey, you will be asked questions about yourself and your IBS diagnosis. This survey should take about 10-15 minutes to complete.

1. What year were you diagnosed with IBS by a health care provider? [enter year]
2. What type of IBS do you have?
   1. Diarrhea predominant
   2. Constipation-predominant
   3. Mixed
   4. Unknown
3. Do you currently suffer from abdominal (stomach) pain? [Yes/No]
   1. If yes, how severe is your abdominal (stomach) pain? [on a 100 point scale]
   2. If yes, please enter the number of days that you get the pain in every ten days. [from 1 – 10 numerical value]
4. Do you currently suffer from abdominal distention(bloating)? [Yes/No]
   1. If yes, how severe is your abdominal distention/tightness? [on a 100 point scale]
5. How satisfied are you with your bowel movements? [on a 100 point scale]
6. How much is your IBS affecting or interfering with your life in general? [on a 100 point scale]
7. Which of the following help you manage your IBS? [select all that apply]
   1. Gastroenterologist
   2. Family physician
   3. Dietician
   4. Pharmacist
   5. Psychologist
   6. Counsellor
   7. Social Worker
   8. Hypnotherapist
   9. Naturopath
   10. Osteopath
   11. Traditional Chinese Medicine (TCM) practitioner
   12. Health Coach
   13. Yoga Instructor
   14. Massage Therapist
   15. Visceral Therapist
   16. Pelvic floor specialist
   17. Support groups and/or other IBS patients
   18. Other [open text]
8. What types of digital tools are you using or have used to manage IBS?
   1. Apps that I have downloaded specific to GI, diet, etc.
      1. Branching question to:
         1. IBS-specific apps
         2. General GI and health apps
         3. Health journalling apps
         4. Diet related apps
         5. Cognitive-behavioural therapy and or gut hypnotherapy apps
         6. Other [open text]
   2. Podcasts related to IBS e.g., food, therapy, microbiome, information, recipes
   3. Apps that track movement e.g., physical activity, daily living activities
   4. Websites
   5. Support groups
9. What is your year of birth? [enter year]
10. What gender do you identify as?
    1. Man
    2. Woman
    3. Non-binary
    4. Transgender
    5. Two-spirit
    6. Other gender not listed above
    7. Prefer not to answer
11. Do you identify as any of the following? Choose all that apply.
    1. Racialized/visible minority
    2. Persons with disabilities
    3. Indigenous
    4. New to Canada (less than 5 years)
    5. Born outside of Canada
    6. 2SLGBTQI+
    7. I have children/grandchildren 18 or under living at home
    8. Other (please specify)
    9. Prefer not to answer
12. What is the highest level of education you have completed?
    1. No certificate, diploma or degree
    2. High (secondary) school diploma or equivalency certificate
    3. Postsecondary certificate or diploma below bachelor level/apprenticeship or trades certificate
    4. Bachelor’s degree
    5. University certificate or diploma above bachelor level
    6. Degree in medicine, dentistry, veterinary medicine or optometry
    7. Master’s degree
    8. Earned doctorate
    9. Prefer not to answer
13. Is there anything else that you would like to share with us? [free-text]

We are looking forward to meeting you during the focus groups. Thank you!
