# Supplementary material for: Navigation, Adoption, and Use of Digital Health Technologies for Irritable Bowel Syndrome Self-Management: Focus Group Study of Patient Experience and Decision-Making
Source: JMIR Hum Factors. 2026 Feb 2;13:e75012. doi: 10.2196/75012 (PMC12910269; doi:10.2196/75012)
Supplement: Multimedia Appendix 3 [file humanfactors_v13i1e75012_app3.doc]

## **Multimedia Appendix 3:** Semi-structured focus group interview guide.

**Focus Group Questions**

We are interested in hearing about your experience as an IBS patient in managing your disease with the support of other support persons, digital tools, and online resources.

We define digital resources as information, tools, materials, and support from online resources and meeting spaces. We define apps as a digital tool downloaded onto your smart phone.

We are hoping to understand how IBS patients are finding resources and what resources have been helpful. We would also like to know why or why not you decide to use a digital tool, and why you continue to use it.

We are using this focus group to complement what we have learned through a literature review. We will take what we learn today and combine it with what we know, to better inform researchers and developers in advancing the quality and accessibility of current and future digital resources for IBS patients.

1. **GENERAL QUESTIONS ON DIGITAL RESOURCES**

***In the survey you completed, many of you have indicated you used these (list) digital resources.***

1. What has been your experience navigating digital resources to self-management your IBS?

Facilitator: Please probe on:

- 1. Where do you go for IBS information?
  2. What resources are you currently using?
  3. How are you currently accessing these resources?
  4. Who, if anyone, recommended the resources?
  5. What do you like/dislike about these resources?
  6. What resources have been helpful? Why or why not?
  7. How do you decide whether a resource is trustworthy or not?
  8. What resources do you currently do not have to that you wish you did?

App-specific probes:

***Based on your survey responses, the most commonly used apps were (list).***

1. How did you decide which app to use or try?
2. What was your experience in incorporating the app into your daily life?
3. What aspects do you like/dislike?
4. What features do you like or find helpful when using the app?
5. How frequently do you use the app? What helped you continue to use the app?
6. If you stopped using the app, why did you do so?
7. What challenges did you have using the app?
8. What improvements would you make to the app(s) you have tried?

**Our research suggests IBS patients are not readily using digital tools. If digital tools are used, they are not consistently used and over a long period of time, despite the abundance of IBS resources available.**

1. What are your thoughts on these findings?

Facilitator: Please probe on:

- 1. Why did you use to decide to use such a tool?
  2. For those who stopped using the tool, why did you decide to do so?
  3. Why do you think IBS patients may not be using digital resources to self-manage?
  4. What may be some factors or considerations that may deter IBS patients form accessing or using these resources?
  5. What would help support you better?

1. **CLOSING QUESTIONS (Last 15 minutes)**
2. Based on our conversation today, what do you think are priorities for improvement when it comes to digital tools for IBS management?
3. Before we conclude, is there something else you would like to add?
