# Supplementary material for: Navigation, Adoption, and Use of Digital Health Technologies for Irritable Bowel Syndrome Self-Management: Focus Group Study of Patient Experience and Decision-Making
Source: JMIR Hum Factors. 2026 Feb 2;13:e75012. doi: 10.2196/75012 (PMC12910269; doi:10.2196/75012)
Supplement: Multimedia Appendix 4 [file humanfactors_v13i1e75012_app4.docx]

**Multimedia Appendix 4:** Participant quotes of observed influences in considering DHT adoption and use for IBS patients in relation to key constructs based on the Expanded Unified Theory of Acceptance and Use of Technology (UTAUT2) model.

| **Key Construct** | **Influence** | **Participant Quotes** |
| --- | --- | --- |
| Performance Expectancy | Perceived effectiveness of the digital health tool; IBS patients’ expectations | *“And I found [the FODMAP-specific mHealth app] really helpful in the beginning, checking food after I had a reaction to something [ . . . ] and narrowing down what was actually probably the cause.” (Participant #43)*  *“This is just an assumption [ . . . ] some people want a medication to fix them. They don't want to have to do the hard work where they're having to do so much of the work themselves, controlling their diet [ . . . ] they just wanted somebody else to fix them, whether it be a medication or whatever the case may be. And that's the approach that they stick with. And it is hard work to manage your diet and to manage all those external factors.” (Participant #29)* |
| Effort Expectancy | User-friendly design; customization; minimal effort to use; accessibility | *“There are lots of tools, but because [the app] is not customizable at this point and not user-friendly in terms of physical activity that you can plan and do, that might be a hindrance for some people if they have to log into a computer, right?” (Participant #9)*  *“I was actually just looking at the app on my phone and I realized I actually did not use it on my phone at all because honestly, the layout on phone is not very user friendly. It's pretty small and I think I'm getting older too, so it's hard to use my phone with the small font size.” (Participant #14)*  *“If they're online, it's ease of access. And again, the exercises you can do in your own home at your own time, whenever you feel like your body wants to exercise. [ . . . ] And the meditation apps or podcasts that I listen to [ . . . ] when you feel stressed, you want to go there and use them.” (Participant #37)* |
| Social Influence | Recommendations from families, friends, peers, and social media groups | *“On Facebook, there's a low FODMAP group that I creep in on occasionally for food and recipe ideas.” (Participant #9)*  *“Friends and family have recommended apps but they have a different focus [ . . . ] Other patients' recommendations and the app store, I'll look at the free apps first - that's my first choice.” (Participant #20)*    *“For me, it's more about the emotional component of IBS. So sometimes I just go to Reddit and see how people are dealing with their symptoms and not for I guess any scientifically backed information, but just emotionally how people deal with their symptoms.” (Participant #14)* |
| Facilitating Conditions | Digital health literacy; computer or mobile competency | *“I found information on medical websites. I found studies and could see the results. I have a background in reading (formal) documentation, so it wasn't that hard to follow along most of it.” (Participant #43)*    *“Could it be different generations and different approach to–my mom, she hates her phone or she hates computer. And for her [ . . . ] it's a significant learning curve.” (Participant #4)* |
| Hedonic Motivation | Desire for knowledge and improvement of IBS | *“I think a key thing is new content - to keep it interesting and if there's new research or something, making sure that it's current. That's really important to me.” (Participant #48)*  *“The apps necessarily weren't engaging, how can I describe this? Yeah, I got bored. That was it. I like to be mentally challenged a lot in my day-to-day. And so it becomes too tedious, like you've mentioned then I haven't got time for such things in my day anymore. It just takes up too much of my time and it's not intellectually stimulating.” (Participant #37)* |
| Price Value | Perceived price value of the digital health tool; associated monetary cost of the DHT | *“A consideration for me is cost as well. If I am looking at an app and it's five bucks or ten bucks, I am not going to try it because that's still five or ten bucks, and I don't know if it's going to work for me or if it's even worthwhile.” (Participant #9)*  *“There are so many others that it is an ongoing monthly fee and that kind of puts me off [ . . . ] if you're sucked into this payment thing, I don't think, I wouldn't give it a good try.” (Participant #48)*  *“[ . . . ] I'll go to the app store and I'll look at the free apps first - that's my first choice. A lot of them [have] a premium, you get a whole bunch of ads and it's free, but then if you pay to upgrade you get more functionality or just better user experience. I'll go free first and try them, and if they really suck I'll end up with something- I don't mind doing a one-time payment [ . . . ] but I will never do a subscription - that's a flat-out no for me, never.” (Participant #20)* |
| Habit | Habit developed from DHT; frequency of DHT use | *“Yeah, I think every once in a while, I look through all the apps on my phone or actually my phone will say ‘you haven't used this for six months’ or whatever. And that's usually an indication that I don't need that one because it isn't useful to me. But the ones that I do stick with are, I feel I'm getting some result from them? Otherwise, it would be a waste of time.” (Participant #48)*  *“I actually really like (the DHT). [ . . . ] It was part of the study, it has some reminders. Reminders to move, which is kind of annoying, but actually good because I realize sometimes I'm sitting for an hour and not moving. So that is actually helpful.” (Participant #14)*  *“I second the fact that my phone is always with me, so having an app is convenient and having a reminder is also convenient. If I have to remember to go to a website and log in, I'll be setting a reminder in my phone maybe with a link to the website, but it's just an extra step.” (Participant #20)* |
| Trust | Perceived likeability and trust in the DHT; willingness to believe in the DHT | *“If I had seen an endorsement from the UofC for any of the apps, I would've jumped all over them just to know that that had been reviewed and vetted by a university, a great medical program [ . . . ] being endorsed by an academic institution specifically with medical program would be important.” (Participant #29)*    *“An app that's recommended by a doctor, I would try.” (Participant #20)*  *“Well, I think you just have to sometimes look at where the source is from [ . . . ] I just wanted good solid information, and [the Mayo Clinic and National Institutes of Health] are the sites that I tended to go for. [ . . . ] They all have their studies attached to the articles that they write, and you can drill into those as well and check those out.” (Participant #43)*  *“I will not take any risk of paying $3,000 for something that somebody online that it would help. The final decision will be after visiting medical establishments and talking to professional.” (Participant #4)* |
| Risk | Conflicting information; scams and exploitation;  transparency concerns | *“I spend a lot of time reviewing terms and conditions, especially at subscriptions for websites and their privacy policies and things like that. And I think being really clear and transparent about that, about their policies and really indicating where's the data being stored, what is it going to be used for, who's going to see it? How is it going to be shared? Those are all really important questions that need to be answered upfront for the patients.” (Participant #29)*  *“[ . . . ] Things that are online, there's too much out there and I think it's really hard to know what's accurate and what's going to be helpful and what to actually focus on.” (Participant #29)*  *“I've never heard about that and I would've think it's a scam. Right?” (Participant #4)* |

**Note***: DHT,* digital health technology; “*trust*” and “*risk*” key constructs were not part of the original UTAUT2 model and were included as a result of the study’s findings.
